# Septic sacroiliitis in children-a diagnostic challenge

**DOI:** 10.1186/1546-0096-9-S1-P233

**Published:** 2011-09-14

**Authors:** N Scotti, G Russo, R Carlomagno, C Forni, F Orlando, MG Della Casa, M Alessio

**Affiliations:** 1 Department of Pediatrics, Federico II University, Naples, Italy

## 

Septic sacroiliitis is rare and accounts for approximately 1-2% of osteoarticular infections in children. Diagnosis of this disease has been difficult in the past due to its deep location and may be delayed due to the lack of specific clinical signs and symptoms. We describe 2 paediatric patients with clinical-radiological signs of septic sacroiliitis. E., an 8-year-old boy, presented with right hip/back pain and pyrexia after physical exertion. Examination disclosed tenderness of the right sacroiliac joint and buttock. Laboratory indicators of infection were elevated. Bone scintigraphy showed increased uptake in the right sacroiliac joint, CT-scan revealed a lytic area, MRI pelvis showing right-sided sacroiliac joint fluid collection with adjacent oedema of the ileum. These findings led to diagnosis of septic sacroiliitis. R. a 5-year-old child, presented with buttock pain after physical stress, with slight fever. She had severe pain on stressing her left sacroiliac joint; increased inflammatory parameters and neutrophilia were detected also. MRI showed left ileum and sacral intraspongious oedema, with ipsilateral iliopsoas and buttock muscles involvement. The diagnosis of septic arthritis was made. The children were managed conservatively with 3 weeks of IV antibiotics and 3 weeks of oral antibiotics and showed good clinical response with no sequelae during follow-up. In conclusion, an early diagnosis of septic sacroiliitis is very important in order to avoid complications and to reserve surgical approach to unresponsive cases. Moreover, it is signalled the value of the physical effort as predisposing factor and the utility of the MRI, both in diagnostic and follow-up.

